# Pharmacometabolomics Informs Viromics toward Precision Medicine

**DOI:** 10.3389/fphar.2016.00411

**Published:** 2016-10-27

**Authors:** Angeliki Balasopoulou, George P. Patrinos, Theodora Katsila

**Affiliations:** ^1^Department of Pharmacy, School of Health Sciences, University of PatrasPatras, Greece; ^2^Department of Pathology, College of Medicine and Health Sciences, United Arab Emirates UniversityAl Ain, United Arab Emirates

**Keywords:** pharmacometagenomics, pharmacometabolomics, pharmacogenomics, precision medicine, viromics, metagenomics

## Abstract

Nowadays, we are experiencing the big data era with the emerging challenge of single data interpretation. Although the advent of high-throughput technologies as well as chemo- and bio-informatics tools presents pan-omics data as the way forward to precision medicine, personalized health care and tailored-made therapeutics can be only envisaged when interindividual variability in response to/toxicity of xenobiotics can be interpreted and thus, predicted. We know that such variability is the net outcome of genetics (host and microbiota) and environmental factors (diet, lifestyle, polypharmacy, and microbiota) and for this, tremendous efforts have been made to clarify key-molecules from correlation to causality to clinical significance. Herein, we focus on the host–microbiome interplay and its direct and indirect impact on efficacy and toxicity of xenobiotics and we inevitably wonder about the role of viruses, as the least acknowledged ones. We present the emerging discipline of pharmacometabolomics-informed viromics, in which pre-dose metabotypes can assist modeling and prediction of interindividual response to/toxicity of xenobiotics. Such features, either alone or in combination with host genetics, can power biomarker discovery so long as the features are variable among patients, stable enough to be of predictive value, and better than pre-existing tools for predicting therapeutic efficacy/toxicity.

## Introduction

**FIGURE 1:**
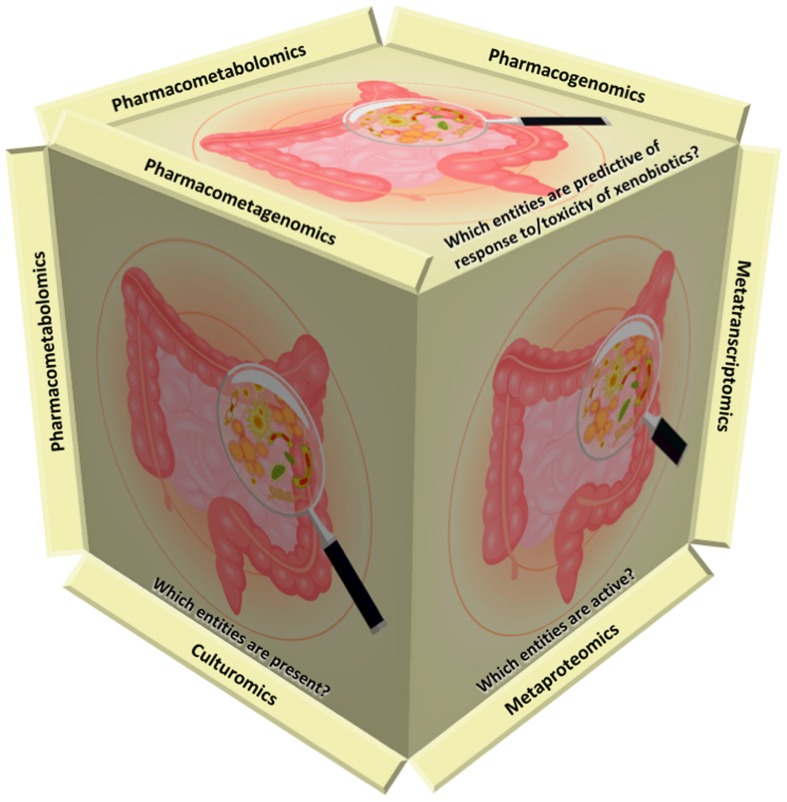
**Omics strategies complement each other to address current challenges.** Metagenomics aims to address the question of “which entities are present?” via high-throughput sequencing or microarrays. Recently, culturomics has been introduced as a complementary approach to metagenomics. Viromics (viral metagenomics) studies aim to identify resident viruses. Metatranscriptomics and metaproteomics focus on “which entities are active?” Pharmacometabolomics (the later term used synonymously with pharmacometabonomics), pharmacogenomics and pharmacometagenomics aim to predict the response to and/or toxicity of xenobiotics on the basis of pre-dose profiling.

Complex interactions between the host immune system and microbiota are of dynamic nature and hence, of fundamental importance when homeostasis is considered, as the host is exposed to trillions of indigenous microorganisms; bacteria, archaea, fungi, and viruses (Hooper et al., 2012; Virgin, 2014) (**Figure 1**). Metagenomics data suggest that the microbiome of healthy humans includes several viral genes and the intestines and skin of healthy individuals are associated with viruses that replicate in eukaryotic and prokaryotic cells (Reyes et al., 2010). Temperate bacteriophages, which are assembled and propagated stably within the bacterial host chromosome, are considered as the predominant viral group (Reyes et al., 2010; Minot et al., 2013). Plant-infecting viruses, herpesviruses, poxviruses and picornaviruses are less abundant and often derived from diet (Kim et al., 2011). Yet, the great majority of viruses remains unidentified (Minot et al., 2012a,b; Reyes et al., 2012) and much less is known about the role of resident viruses.

We believe in a systems-level understanding of the host–microbiome interplay and its impact on the efficacy/toxicity of xenobiotics with an emphasis on resident viruses, as their diversity, abundance and role is still poorly understood. Herein, we wonder about the role of viruses, as the least acknowledged ones and we present the emerging discipline of pharmacometabolomics-informed viromics, in which pre-dose metabotypes (metabolic phenotypes) can assist modeling and prediction of interindividual variability in response to/toxicity of xenobiotics. Such features, either alone or in combination with host genetics, can power biomarker discovery so long as the features are variable among patients, stable enough to be of predictive value, and better than pre-existing tools for predicting therapeutic efficacy/toxicity.

### Horizontal Genetic Transfer Results in Evolutionary Changes and Confers Interindividual Variability

Viral–microbial interactions in the human gut seem to be rather different from the predator-prey relationship that is known as “kill the winner” and dominates in other ecosystems (Reyes et al., 2010). Horizontal genetic transfer occurs frequently among gut microbes (Roberts et al., 2008; Tamames and Moya, 2008). Viruses, particularly bacteriophages, are one of the main drivers of the evolutionary change seen in microorganisms through horizontal genetic transfer. The observation that gut microbial metagenomes consist of a large number of phage-related genes implies a viral role in gut homeostasis (Qin et al., 2010). In healthy gut, viruses may elicit innate immune responses. Pattern-recognition receptors can detect viral components (Takeuchi and Akira, 2010) and initiate the crosstalk between resident viruses and the innate immune system. Commensal bacteria-depleted mice have been reported to recover from severe gut inflammation upon administration of lipoteichoic acid or lipopolysaccharide, implying that toll-like receptors have a protective role that extends beyond the recognition of commensal bacteria to other microbes (Rakoff-Nahoum et al., 2004). Resident gut viruses have reduced inflammation via toll-like receptor 3- and 7-mediated interferon-beta production, shedding light for the first time on the interplay of host innate immune system and viruses (Yang et al., 2016). Horizontal genetic transfer confers increased pathogenicity, antibiotic resistance and new metabolic activity (Reyes et al., 2010; Minot et al., 2011). Therefore, bacteriophages are of biomedical importance.

### Within-Individual and Inter-individual Viral Evolution

Metagenomic data emphasize that bacterial strains are significantly different among individuals, although their gut typically contains bacteria from only a few phyla (Turnbaugh et al., 2009). Different body sites are also inhabited by different bacterial strains (Peterson et al., 2009; Blaser, 2010; Chen et al., 2010; Qin et al., 2010). This variation may account for their highly variable phage predators, as phages can be highly selective (Rodriguez-Valera et al., 2009). Notably, phage sensitivity is used in the clinic to distinguish bacterial strains (Sell et al., 1983; Mahony et al., 1991). Furthermore, the great variability reported in phage populations among individuals may come from within-individual viral evolution, when long-term viral residents are considered. Rapid within-host viral evolution has been reported, suggesting that multiple new viral species arise in the gut of a typical human over the course of human life (Minot et al., 2013). Notwithstanding, the forces diversifying bacteriophage genomes in human hosts have not been studied in detail.

We strongly feel that changes in abundance and composition of microbiota (dysbiosis) underline interindividual variability. That said, gut microbiota and particularly, resident viruses have not been a focus for the drug metabolism and toxicology communities, despite several early studies showing their importance in xenobiotic biotransformation (Boxenbaum et al., 1979; Illing, 1981). Notwithstanding, re-evaluation and awareness are critical, as microbiota represent a source of physiological variability among individuals and populations that can readily affect the disposition and toxicity of xenobiotics and their metabolites. These effects can be direct or indirect (Nayak and Turnbaugh, 2016). Indirect effects include the metabolic exchange and the co-metabolism and processing of endogenous and dietary substrates (Nicholson and Wilson, 2003). Gut microbiome can alter the expression of host’s pharmacogenes (Björkholm et al., 2009). Microbiome-derived metabolites have been reported to modulate the drug metabolizing systems of the host (Wilson and Nicholson, 2016). Orally administered xenobiotics are exposed to gut microbes prior to their absorption and hence, their bioavailability and half-life is altered (Al-Hilal et al., 2013). The compositional and/or functional alterations in gut microbiota brought about by polypharmacy or the administration of antibiotics, probiotics, or prebiotics greatly increase interindividual variability in response to xenobiotics (Li and Jia, 2013). Notably, the role of resident viruses is still poorly understood.

## Challenges and Opportunities

If microbiome in total has escaped our full attention so far, resident viruses in humans and animals are poorly acknowledged today. Even though viruses are extremely important to the ecology (Roossinck, 2015; Puxty et al., 2016), the discipline of viromics (or viral metagenomics, as it is used interchangeably) can be currently considered as the most elusive of the -omics fields.

## “Which Entities are Present?”

The human body is a host for a complex living microbial community consisting of bacteria, archaea, fungi, protozoa and viruses, which together constitute the human microbiota. The total genome of microorganisms is also the human metagenome and hence, metagenomic studies aim to address the question of “which entities are present?” via high-throughput sequencing or microarrays. Technical challenges are still profound, when seeking for an answer to that question (**Figure 1**).

Viruses outnumber microbial cells 10:1 in most environments, yet, viral DNA only represents 0.1% of the total DNA in a microbial community, such as the human gut (Qin et al., 2010). If a deep sequence coverage of the human viruses is desired, viral particles should be isolated (Reyes et al., 2010; Hurwitz et al., 2013). As the amount of the nucleic acids extracted from purified viral particles is often below the required threshold for sequencing, several amplification methods have been developed; random amplified shotgun library (Rohwer et al., 2001), linker-amplified shotgun library (Breitbart et al., 2003) and multiple displacement amplification (Hutchison et al., 2005) are a few examples. The latter method is greatly advantageous, as it allows the amplification of complete viral genomes, although recent reports have implied that critical biases and contamination is an issue (Zhang et al., 2006; Yilmaz et al., 2010).

Today, the majority of the viral sequences are novel or enriched in regions of low-complexity repeats. Thus, sequencing technologies that prioritize long-read lengths over those of short-read lengths are preferred (Wommack et al., 2008). However, sequencing technologies of long-read lengths, such as 454/Roche pyrosequencing, are about to be discontinued (Bikel et al., 2015). Bioinformatics tools that have been developed to analyze viruses from short sequence reads have accepted this challenge (Liu et al., 2007). Moreover, the majority (usually, 60–99%) of the viral sequences studied so far have no significant similarity to other sequences in databases or have higher homology to prokaryotic or eukaryotic genes (Breitbart et al., 2002, 2003; Blomström et al., 2010). A crucial step in viromics is the filtering of bad quality sequences or the decontamination of 16S rRNA, 18S rRNA and human sequences by mapping. Mapping algorithms or tools, such as ViroBLAST (Deng et al., 2007), BLASTX (Segata et al., 2012) or USEARCH (Edgar, 2010) are employed to obtain the taxonomic composition of a viral community. Taxonomic and functional assignments are crucial for the viral community profile to be created, since it reflects sample diversity. Today, the number of the deposited genomes in databases is far less than the expected number of virotypes (Rohwer and Thurber, 2009), while most of the new sequences are poorly annotated (Brister et al., 2014; Martınez et al., 2014). The percentage of sequence reads with similarity to known viral sequences depends on the database used and on how well sequences have been filtered. This percentage is less than 0.01% (Yang et al., 2011; Bikel et al., 2015). MetaVir, VIROME, and iVirus are few publicly available databases (Sullivan, 2015).

To assist viromics data analysis similarity-independent methods have been also developed. PHACCS assesses the biodiversity of uncultured viral communities and quantifies virotypes (Breitbart et al., 2002; Angly et al., 2005) and CRASS allows the simultaneous cross-assembly of all the samples in a dataset to identify shared viruses (Dutilh et al., 2012). MaxiPhi uses pairwise assemblies from pooled viromes (Angly et al., 2005). To tackle chimeras, the overlap–layout–consensus algorithms have achieved the assembly of viral genomes. Newbler has been extensively used in viral and bacterial shotgun metagenomic projects (Qin et al., 2010; Reyes et al., 2010; Bikel et al., 2015). Yet, it remains to be determined, if Newbler will be discontinued with the 454/Roche in 2016 (Bikel et al., 2015). Minimo is designed for the assembly of small datasets (Treangen et al., 2011) and VICUNA is an assembler that is specialized in *de novo* assembly of data from heterogeneous viral populations (Yang et al., 2012). MetaVelvet (Namiki et al., 2012) and other de Brujin graph assemblers are an alternative to the overlap–layout–consensus assemblers and have also been used on the assembly of viral genomes (Hurwitz and Sullivan, 2013). Sequencing of total RNA viruses has been proven impractical (Robertson et al., 2010).

## “Which Entities are Active?”

One might have already thought that this question is even more difficult to address. We share an optimistic view on the basis of the metatranscriptomic, metaproteomic and metabonomic studies being reported (Lim et al., 2013; Aguiar-Pulido et al., 2016; Yu et al., 2016). The NIH-sponsored Human Microbiome Project has been established to comprehensively characterize human microbiota from multiple body sites and analyze their impact in human health and disease (Peterson et al., 2009; Proctor, 2016). The Vaginal Human Microbiome Project has validated a protocol that achieves species-level classification of V1–V3 16S rRNA sequence from the vaginal microbiome (Fettweis et al., 2012). Culturomics, coupled to matrix-assisted laser desorption/ionization time-of-flight (MALDI-TOF) mass spectrometry, has been also introduced as complementary to metagenomics to study complex microbial ecosystems, such as the human gut. Lagier et al. (2012) provided a proof-of-concept applying 212 different culture conditions and successfully culturing 340 different bacterial species, five fungi and for the first time, Senegalvirus (Greub, 2012; Lagier et al., 2012). Dubourg et al. (2014) coupled culturomics with pyrosequencing to address the effect of antibiotics in gut microbiota diversity (Dubourg et al., 2014). Yet, resident viruses and their interplay with the host (human or other microbes) are poorly studied.

## “Which Entities are Predictive of Response to/Toxicity of Xenobiotics?”

Every human being is unique and microbiota that colonize the human body shape individuality to a great extent. Interindividual variability is a prerequisite to tailored-made therapeutics and personalized health care. A systems-level understanding is required to delineate the host–microbiota interplay. The advent of high-throughput technologies and bio- and chemo-informatics tools presents pan-omics approaches as the way forward. Can we predict xenobiotics efficacy/toxicity by pre-dose profiling?

Traditionally, the host has been our focus, embracing the potential of pharmacogenomics and pharmacometabolomics. Pharmacogenomics has pioneered the prediction of the outcome of a xenobiotic intervention in an individual based on an analysis of that individual’s genetic profile (Smalley and Sondak, 2010; Ritchie, 2012; Hershfield et al., 2013; Everett, 2015). Today, FDA-approved drug labeling may contain information on pharmacogenomic biomarkers. The U.S. National Human Genome Research Institute and the National Academy of Medicine recently brought together 25 innovative genomic medicine programs to coalesce innovative genomic medicine programs around concrete and at the same time, compelling signature projects accelerating the responsible implementation of genomic medicine in efforts to improve clinical care worldwide (Manolio et al., 2015). Pharmacometagenomics are anticipated to perform an analogous task to that of pharmacogenomics focusing on human metagenome (the total genome of microorganisms). Indeed, a more mechanistic understanding of which microbes and genes contribute to xenobiotics efficacy/toxicity may enable prediction of which patients will derive greatest benefit from a therapeutic intervention (Haiser et al., 2013; Nayak and Turnbaugh, 2016). Pharmacometabolomics is predictive of the outcome of a xenobiotic intervention in an individual based on a mathematical model of pre-intervention metabolite signatures (Nicholson et al., 1999; Clayton et al., 2006). Pharmacometabolomics is based on metabotypes, as they are the net result of genetic, physiological, chemical, and environmental influences (Holmes et al., 2008; Everett, 2015). Recently, a new concept has arisen, namely “pharmacometabolomics-aided pharmacogenomics,” to reinforce the identification and validation of clinically relevant associations (Ji et al., 2011; Suhre et al., 2011; Abo et al., 2012). We have recently proposed that pharmacometabolomics-aided pharmacogenomics may have an even greater impact if coupled to information technologies to facilitate data analysis and sense- and decision-making on the basis of a synergy between artificial and human intelligence (Katsila et al., 2016).

If we want to turn information growth into knowledge growth and better informed decisions, the implementation of new working practices is more than necessary. A paradigm may well be pharmacometabolomics-informed viromics. In an analogy to pharmacometabolomics-aided pharmacogenomics, pharmacometabolomics-informed viromics benefits from pre-dose metabotypes that can assist modeling and prediction of interindividual response to and/or toxicity of xenobiotics, this time not focusing on the host, but on the resident viruses and their interactions. Although bacteria have been directly associated with dysbiosis and interindividual variability upon xenobiotics administration, the role of the virome in the microbial community should be further explored. So far, scientists have been focusing on the “which entities are present?” and “which entities are active?” questions with the aim to address the “which entities are predictive of response to/toxicity of xenobiotics?” challenge. Instead, we suggest a global focus on metabolites as the biochemical end points of complex interactions between the host and its environment (including the gut microbiome). Thus, a global readout will be revealed mapping microbial, viral and mammalian interindividual variability upon xenobiotics administration. Our next step is to integrate phenotypic and genotyping approaches with information technologies.

**FIGURE 2:**
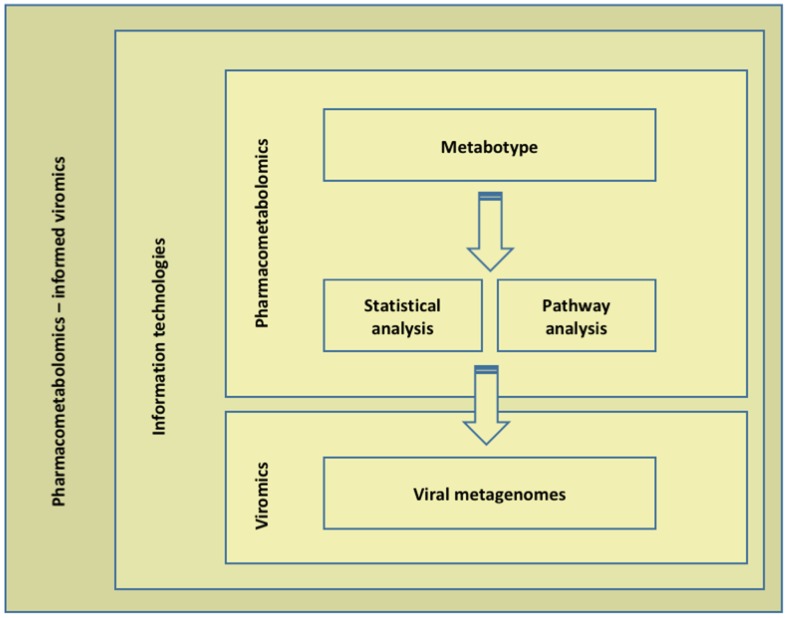
**A pharmacometabolomics-informed viromics workflow – the overall multilayered strategy.** Instead of any single omics approach, we propose an integrated (transomic) analysis. In this context, we have two layers of systems-scale molecular measurements; the pharmacometabolome (layer 1) and the virome (layer 2). Layers 1 and 2 are coupled to information technologies.

Instead of any single omics approach, we propose an integrated (transomic) analysis that is anticipated to provide more insights into the emergence of the phenotypes in question (response to/toxicity of xenobiotics) than any layer can by itself, highlighting the complementarity of a multilayered strategy. In this context, we have two layers of systems-scale molecular measurements; the pharmacometabolome (layer 1) and the virome (layer 2; **Figure 2**). Layer 1 characterization includes sample acquisition and preparation, analysis (NMR or mass spectrometry technologies), data processing and data analysis (targeted and untargeted). Particularly, untargeted analysis is of great benefit as a tool to shape hypothesis; multiple analytes are quantified simultaneously and pharmacometabolomic modeling is not limited by prior understanding or hypotheses. Such metabotype-based findings may be patient and/or xenobiotic profiling. MALDI-TOF mass spectrometry has recently enabled spatial information. Dorrestein’s methodologies aim to scan microbial communities, capture their exact image and identify what microorganisms do in their complex communities by identifying and locating the metabolites produced (Tullis, 2016). Pirhaji et al. (2016) reported PIUMet, a network-based approach, prize-collecting Steiner forest algorithm for integrative analysis of untargeted metabolomics (Pirhaji et al., 2016). PIUMet infers molecular pathways and components via integrative analysis of metabolite features, without requiring their identification. If a hypothesis is already in place, targeted pharmacometabolomics is advantageous (Williams et al., 2016). Then, viromics (layer 2) is performed as of today. VirCapSeq-VERT is ideally suited for the analysis of virome composition and dynamics, as its highly multiplexed nature allows the simultaneous identification and comprehensive genetic characterization of all known vertebrate viruses and their genetic variants as well as the novel ones (Briese et al., 2015). Similarly, quantitative temporal viromics are applied to a wide range of viruses (Weekes et al., 2014). At this point, transomic data sets are generated that consist of pharmacometabolomics (layer 1) and viromics (layer 2) data. We propose in-depth data mining, analysis and argumentation, according to which information technologies provide the means for filtering and systems-level dynamic parameters from fewer samples across broad molecular interaction networks. Data mining, analysis, collaboration and decision-making in such diverse data-intensive and cognitively complex settings is performed via the Dicode approach, supporting artificial and human intelligence (the Dicode platform and services provide a remedy to the information and cognitive overload as users can customize the Dicode workbench via a proper assembly of tools that suit their needs and properly structured data lead to more informed decisions). The envisioned architecture combines batch and stream processing (Karacapilidis, 2013; Tsiliki et al., 2014). Current limitations, such as lack of annotation, lack of a conserved region in the virome and/or “non-cultivable” viral entities are by-passed. Viral–microbiota–host–xenobiotics relationships are revealed and modeled following a multilayered approach.

## Conclusion and Future Perspectives

Microbiota in humans is a collection of microscopic organisms that inhabit the body and contains representatives from all the domains of life: archaea, bacteria, eukarya, viruses. If interindividual variability in response to and/or toxicity of xenobiotics is to be acknowledged, viral–microbial–host–xenobiotics dynamics need to be further clarified. Pharmacometabolomics-informed viromics coupled to information technologies highlight the complementarity of a multilayered approach to turn information growth into knowledge growth.

## Author Contributions

Conception and design: AB, TK, and GP. Writing, review, and/or revision of the manuscript: AB, TK, and GP.
